# Supplementary material for: The Preventive Effect of A Magnetic Nanoparticle-Modified Root Canal Sealer on Persistent Apical Periodontitis
Source: Int J Mol Sci. 2022 Oct 28;23(21):13137. doi: 10.3390/ijms232113137 (PMC9655100; doi:10.3390/ijms232113137)
Supplement: Supplementary file 1 [file ijms-23-13137-s001.zip › ijms-1968202-supplementary.pdf]

The preventive effect of a magnetic nanoparticle-modified root canal sealer on persistent apical periodontitis

Xiao Guo [1,2,†], Yan Sun [1,2,†], Zheng Wang [1,2], Biao Ren [1], Hockin H. K. Xu [3], Xian Peng [1], Mingyun Li [1], Suping Wang [4], Haohao Wang [1,2], Yao Wu [5], Michael D. Weir [3], Xuedong Zhou [1,2], Fang Lan [5,*] and Lei Cheng [1,2,*]

Materials and methods

Apical sealing ability

After 7 days, the root surfaces of all specimens were coated with two layers of nail varnish with the exception of the apical 1 mm. Then immersed in 2% methylene blue solution for 7 days at 37 °C. The specimens were washed with water and dried, and the nail varnish were removed. The samples were then demineralized in 5% nitric acid for 72 h and the solution was changed every 24 h. The specimens were dehydrated in ethanol (80%, 90% ,100%) for 24h respectively. Linear dye penetration was determined using a stereomicroscope with electronic Vernier caliper.

Root preparation and obstruction

A standard access preparation was performed for each tooth. The working length (WL) was established with a no.10 K-file. The file was introduced into the canal until it was visible at the apical foramen, and its length was measured. The WL was determined by subtracting 0.5 mm from this length. Root canals were instrumented by S3 and irrigated with 5.25%NaOCl during the preparation procedures. Final irrigation was performed with 1 ml of 17% EDTA and distilled water. The root canals were dried with sterile paperpoints. Specimens were divided into six experimental groups randomly (five teeth for apical sealing test, five teeth for confocal laser scanning microscopy). The manufacturers' instructions were followed while mixing the root canal sealers, and 0.1% fluorescent Rhodamine B dye was added to sealers for confocal microscopy analysis. The root canals were obturated with sealer and gutta-percha using the cold lateral condensation technique. After obturation, two circular magnets (10 mm in diameter and 5 mm in thickness) were placed away from the sample on the buccal and lingual sides of the sample 5 mm for 5 min. The access openings were closed with light-cure flowable resin and all specimens were kept in 100% humidity at 37 °C for 7 days to allow for the sealer to set.

Colony-forming units (CFU) and crystal violet assay

For CFU counts, specimens were rinsed twice with PBS to remove loose planktonic bacteria. The biofilms were harvested by scraping and vortexing in PBS buffer. After serial dilutions in PBS, the bacteria were incubated on the BHI agar plates to count microorganism colonies and assess the viability.

The specimens were rinsed by PBS twice, then transferred to new 24-well plates and fixed by 100% methyl alcohol for 15 min. Then, specimens were rinsed by PBS and stained with 1 mL 0.1% crystal violet solution for 5 min, then washed with sterile distilled water to remove the residual dye. The bounded crystal violet was released by 1mL 95% ethanol with shaking for 30 min and 200 μL ethanol was transferred to a new 96-well plates. The absorbance of released crystal violet in ethanol was recorded at OD_{595} nm by a spectrophotometer.

Biofilm Imaging

The specimens were gently washed twice with PBS to remove loose planktonic bacteria and fixed with 2.5% glutaraldehyde overnight. Then, the discs were washed twice in sterile water (immersion in water per washed for 10 mins) and serial dehydrated with graded ethanol (50%, 70%, 75%, 80%, 85%, 90%, 95%, and 100%). Then the samples were

sputter-coated with gold for scanning electronic microscopy (SEM) imaging (Quanta 200, FEI, Hillsboro, OR, USA).

For live/dead imaging, biofilms were stained following the manufacturer's instruction (Invitrogen, USA). Briefly, the biofilms were washed with PBS for three times and stained with 2.5 μM SYTO 9 and propidium iodide for 15 min. The samples were imaged with a confocal laser scanning microscope (CLSM) (Olympus FV3000, Japan) equipped with a 60×oil immersion objective lens. The quantification of live/dead was performed and three-dimensional reconstruction of the image with Imaris 7.0.0. Each specimen was scanned in at least five randomly selected positions.

DNA isolation and quantitative real-time polymerase chain reaction

The bacteria were lysed using enzymatic lysis buffer (20 mM Tris-HCl, pH 8.0; 2 mM sodium EDTA and 1.2% Triton X-100) containing 25 mg/mL of lysozyme at 37 °C for 1.5 h. Total DNA of biofilms were extracted and purified using a TIANamp Bacteria DNA kit (TIANGEN, Beijing, China) followed by the manufacturer's directions. The purity and concentration of DNA were detected by NanoDrop 2000 spectrophotometer (Thermo Scientific, USA). The quantitative polymerase chain reaction (qPCR) was used to quantify the number of *E. faecalis, S. gordonii, L. acidophilus* and *A. naeslundii*. The qPCR amplification was performed on the Light Cycler 480 System (Light Cycler 480 System，Roche， Switzerland). The reaction mixture (25 μL) contained Premix Ex Taq (Takara Bio Inc, Shiga, Japan), template DNA, forward and reverse primers (250 NM each). The sequences of the primers for the four bacteria species can be found listed in Table 2. Total genomic DNA samples were extracted using the OMEGA Soil DNA Kit (M5635-02) (Omega Bio-Tek, Norcross, GA, USA), following the manufacturer's instructions, and the quantity and quality of extracted DNAs were measured using a NanoDrop NC2000 spectrophotometer (Thermo Fisher Scientific, Waltham, MA, USA) and agarose gel electrophoresis, respectively. PCR amplification of the nearly full-length bacterial 16S rRNA genes was performed using the forward primer 27F (5′-AGAGTTTGATCMTGGCTCAG-3′) and the reverse primer 1492R (5′-ACCTTGTTACGACTT-3′). The extracted DNA was amplified with two-step PCR, with sample-specific 16-bp barcodes were incorporated into the forward and reverse primers for multiplex sequencing in the second PCR step. Both the two steps of the PCR components contained 5 μl of Q5 reaction buffer (5×), 5 μl of Q5 High-Fidelity GC buffer (5×), 0.25 μl of Q5 High-Fidelity DNA Polymerase (5U/μl), 2 μl (2.5 mM) of dNTPs, 1 μl (10 uM) of each Forward and Reverse primer, 2 μl of DNA Template, and 8.75 μl of ddH2O. Thermal cycling consisted of initial denaturation at 98 °C for 2 min, followed by 25/10 cycles (for first and second amplification step, respectively) consisting of denaturation at 98 °C for 30 s, annealing at 55 °C for 30 s, and extension at 72 °C for 90 s, with a final extension of 5 min at 72 °C. A total of PCR amplicons were purified with Agencourt AMPure Beads (Beckman Coulter, Indianapolis, IN) and quantified using the PicoGreen dsDNA Assay Kit (Invitrogen, Carlsbad, CA, USA). After the individual quantification step, amplicons were pooled in equal amounts, and Single Molecule Real Time (SMRT) sequencing technology was performed using the PacBio Sequel platform at Shanghai Personal Biotechnology Co., Ltd. (Shanghai, China). All raw sequences were deposited in the NCBI Sequence Read Archive under accession number PRJNA787078.

Table S1. Specific Primers used for qPCR.

Bacteria	Template strand
E. faecalis	F：ATTGGAAAGAGGAGTGGCGG<br>R：TGAGCCGTTACCTCACCAAC
S. gordonii	F：GAGTGCTAGGTGTTAGGCCC<br>R：CCTGGTAAGGTTCTTCGCGT
A. naeslundii	F：CTCGACACCGTGAAGTTGGA<br>R：CGACTTCGTCCCAATCACCA
L. acidophilus	F：TGGGGAACCTGCCCCATAG

	R：GGTAGGCCGTTACCCTACCA

The inflammation grade scoring criteria

Inflammatory infiltrate: Dense infiltration zone was defined as an area with intense inflammatory cells and collagen degradation. Normal (score 1), mild (area of dense infiltration zone in periapical lesion <30%, score 2), moderate (area of dense infiltration zone in periapical lesion > 30% and <50%, score 3), severe (area of dense infiltration zone in periapical lesion >50%, score 4);

Thickness of periodontal ligament: Normal (score 1), slightly increased (the widest periodontal ligament space is within 3 times of the normal periodontal ligament thickness, score 2), moderately increased (the widest periodontal ligament space is 3-5 times of the normal periodontal ligament thickness, score 3), severely increased (the widest periodontal ligament space is more than 5 times of the normal periodontal ligament thickness, score 4);

Resorption of mineralized tissues: Absent (score 1), present (score 2).
